# Citrate-Assisted Regulation of Protein Stability and Secretability from Synthetic Amyloids

**DOI:** 10.1021/acsami.4c20784

**Published:** 2025-02-26

**Authors:** Hèctor López-Laguna, Marianna T.P. Favaro, Sara Chellou-Bakkali, Eric Voltà-Durán, Eloi Parladé, Julieta Sánchez, José Luis Corchero, Ugutz Unzueta, Antonio Villaverde, Esther Vázquez

**Affiliations:** † Institut de Biotecnologia i de Biomedicina, 16719 Universitat Autònoma de Barcelona, Bellaterra 08193, Spain; ‡ CIBER de Bioingeniería, Biomateriales y Nanomedicina (CIBER-BBN, ISCIII), 195999 Universitat Autònoma de Barcelona, Bellaterra 08193, Spain; § Departament de Genètica i de Microbiologia, Universitat Autònoma de Barcelona, Bellaterra 08193, Spain; ∥ Departamento de Química, Cátedra de Química Biológica, Facultad de Ciencias Exactas, Físicas y Naturales, ICTA, Universidad Nacional de Córdoba, Av. Vélez Sársfield 1611, Córdoba 5016, Argentina; ⊥ Instituto de Investigaciones Biológicas y Tecnológicas (IIByT), CONICET-Universidad Nacional de Córdoba, Córdoba 5016, Argentina; # Institut de Recerca Sant Pau (IR SANT PAU), Barcelona 08041, Spain

**Keywords:** recombinant proteins, biomaterials, protein secretion, endocrine-like function, cytotoxic proteins

## Abstract

The mammalian endocrine system uses functional amyloids as dynamic depots to store and release protein hormones into the bloodstream. Such depots, acting as secretory granules within the microscale, are formed in specialized cells by the coordination between the ionic, divalent form of zinc (Zn^2+^) and the imidazole ring from accessible His residues. The reversibility of such cross-linking events allows for the release of monomeric or oligomeric forms of the functional protein for biological activity. In vitro, and mimicking such a natural coordination process, synthetic amyloidal granules with secretory properties can be fabricated using selected therapeutic proteins as building blocks. Then, these microparticles act as delivery systems for endocrine-like, sustained protein release, with proven applicability in vaccinology, cancer therapy, regenerative medicine, and as antimicrobial agents. While the temporal profile in which the protein is leaked from the material might be highly relevant to clinically oriented applications, the fine control of such parameters remains unclear. We have explored here how the kinetics of protein release can be regulated by intervening in the storage formulation of the granules, through the concentration of citrate not only as a buffer component and protein stabilizer but also as a chelating agent. The citrate-assisted, time-prolonged regulatable release of proteins, in their functional form, opens a spectrum of possibilities to adjust the preparation of synthetic secretory granules to specific clinical needs.

## Graphical abstract

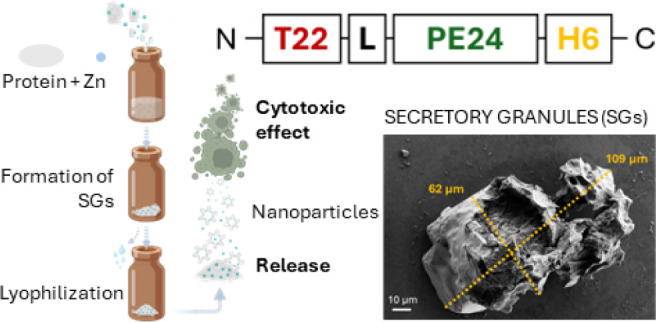

## 1 Introduction

New materials, strategies, and mechanistic approaches for drug delivery are under continuous development,^1–3^ since the current administration protocols, especially at the systemic level, do not always fulfill the required local doses. In particular, slow-release systems^3,4^ might be particularly convenient for a set of chronic conditions for which steady or near-steady drug levels are required, or when the peak-and-trough drug oscillatory patterns, resulting from repeated administration and fast clearance, may pose toxicological or efficacy concerns.^5,6^ In the mammalian endocrine system, intracellular secretory granules (SGs), in between the submicron and micron scales (meaning particles larger than 100 nm but smaller than 1 μm), ensure steady levels of hormones in the blood^7,8^ through regulated, protein release processes. These protein clusters, present in different types of specialized cells, are categorized as nontoxic functional amyloids,^9–11^ and they act as protein storage platforms. In them, protein or peptidic hormones are packaged through reversible cross-molecular interactions mediated by the coordination of Zn^2+^ and solvent-exposed histidine residues.^7,12,13^ Being dynamic depots, they are constructed by protein aggregation in the cell cytoplasm and further growth and condensation or maturation of the granule. Reactive to proper signaling, these granules are released to the cell milieu through the Golgi membranous system for further disintegration (associated with the dilution, displacement, or chelation of Zn^14^) and bloodstream circulation of the detached building block polypeptides.^7^

Aiming at developing novel but methodologically simple platforms for sustained protein drug delivery and inspired by endocrine SGs, we have developed synthetic versions of such protein depots, within the microscale, by the in vitro Zn-assisted aggregation of His-tagged proteins.^15,16^ This is achieved by the addition, in solvent-exposed sites of a selected protein, of hexahistidine segments (H6) through which divalent cations bind simultaneously to more than one single polypeptide, inducing the reversible formation of progressively complex structures, namely nanometric oligomers and micrometric particles.^17^ The H6 tag is necessary for the formation of both, as specific protein species are only aggregated by cationic Zn when H6 is present.^18^ Importantly, the oligomeric intermediates are disassembled by soluble histidine but not by other amino acids,^19^ supporting again the pivotal role of His-rich tags in the assembly process. The molecular mechanisms of Zn/His-mediated protein assembly as SGs and disassembly have been extensively revised elsewhere.^20,21^

In our hands, these protein particles, resulting from mixtures of protein and Zn salts at appropriate ratios, can be formed by structurally different H6-tagged proteins, such as enzymes (the tetrameric E. coli β-galactosidase)^22^ and a wide spectrum of therapeutic proteins. Several types of SGs with clinical interest have been fabricated and successfully tested in cancer therapy (the Pseudomonas aeruginosa exotoxin PE24),^16^ vaccinology (SARS-CoV-2 spike protein and African swine fever virus p30),^23,24^ regenerative medicine (the fibroblast growth factor-2),^25,26^ and as antibacterial agents (GFP fusions to GWH1, T22, Pt5, and PaD),^27^ among many others.^17,28,29^ Showing an amyloidal architecture and being mechanically stable,^16^ synthetic SGs leak the protein building blocks in functional forms, in vitro, under physiological conditions, or in vivo upon subcutaneous administration. This occurs by the progressive disintegration of the material that releases the disassembled components to the media. The fact that these materials are not toxic^30^ and can be stored long-term by lyophilization and further reconstituted before use^31^ makes them suited for industrial-scale production, thus envisaging transference to the Pharma sector and becoming eligible for clinical routes.

An important point regarding the tailoring of this artificial platform for specific delivery purposes is to control the release kinetics. While extremely slow leakage may result in insufficient drug levels, a fast disintegration of the granule may provoke undesired drug toxicities, moving just a little bit beyond the conventional one-shot administration and the consequent peak-and-trough pattern. Using divalent cationic forms of metals alternative to Zn, such as Ca and others,^18^ to induce protein aggregation, distinguishable protein leakage kinetics have been observed.^32^ However, the limitation in the suited clustering elements with divalent ionic forms, that need to be active in protein coordination within the range of recommended dietary doses (to prevent toxicities), makes the use of alternative clustering agents a nongeneric approach, as it results in discrete element-linked kinetics but not in a continuous regulatory potential of the delivery platform. Also, alternative metals might influence the therapeutic outcomes linked to biological side effects of a particular divalent cation. In this context and by taking ionic Zn as a reference molecular glue (as it seems to be a universal protein clustering agent in natural functional amyloids),^13,14,33^ we have explored how to reach regulatable protein leakage profiles through modifying the concentrations of citrate, one of the components of the granule’s resuspension buffer.^31^ By doing so, protein leakage can be finely regulated in a continuous manner, paving the way for the smooth adaptation of the platform to different requirements of temporal protein availability demanded by specific clinical settings.

## 2 Experimental Section

### 2.1 Protein, and Protein Production and Purification

The protein selected as a model for the study (T22-PE24-H6) is a tumor-targeted version of the exotoxin from Pseudomonas aeruginosa. The construct carries the peptide T22, an amino-terminal peptide that specifically interacts with the cell-surface cytokine CXCR4,^34,35^ overexpressed in a plethora of cancer types.^36^ PE24 catalyzes the ADP-ribosylation of elongation factor 2 (EF-2), leading to irreversible inhibition of protein synthesis and subsequent cell death.^37^ An H6 tag, incorporated near the carboxy terminus of the protein sequence, ensures efficient purification, facilitates Zn^2+^ coordination, and promotes subsequent protein clustering. The KDEL peptide, necessary for the cytotoxic activity of PE, is placed at the C-terminus to preserve such cytotoxic activity and to allow for the consequent antitumor effect of the construct^38^

The Escherichia coli codon-optimized gene encoding T22-PE24-H6 was provided by Gene-Art (ThermoFisher), as subcloned into a pET22b plasmid, which was transformed into E. coli Origami B (BL21, OmpT^–^, Lon^–^, TrxB, Gor^–^; Novagen). Gene expression was carried out at 20 °C overnight upon the induction of gene expression with 0.1 mM isopropyl-β-d-thiogalactopyranoside (IPTG), followed by bacterial cell harvesting by centrifugation (15 min, 5,000 × *g*). For protein purification, cells were resuspended in Wash buffer (20 mM Tris, 500 mM NaCl, 10 mM Imidazole, pH 8) in the presence of protease inhibitors (cOmplete EDTA-Free, Roche), being submitted to three rounds of disruption (Emulsiflex-C5 Homogenizer; Avestin) at 500–1000 psi. The soluble fraction was separated by centrifugation (45 min, 15,000 × *g*) and purification was performed by Immobilized Metal Affinity Chromatography (IMAC) using HisTrap HP 5 mL columns in a ÄKTA pure system (Cytiva). Elution was achieved by an imidazole gradient in elution buffer (20 mM Tris, 500 mM NaCl, 500 mM Imidazole, pH 8), and eluted proteins were finally dialyzed against a sodium hydrogen carbonate solution (166 mM NaHCO_3_, pH 8). Protein purity was assessed by sodium dodecyl sulfate polyacrylamide gel electrophoresis (SDS-PAGE), and western blot immunodetection used an anti-H6 monoclonal antibody (Santa Cruz Biotechnology). Protein integrity was analyzed by matrix-assisted laser desorption ionization time-of-flight (MALDI-TOF) mass spectrometry. Final protein concentration was determined by Bradford assay (Bio-Rad).

### 2.2 In Silico Prediction of Protein Structure

The three-dimensional (3D) structure of the folded protein state was computationally predicted using the ColabFold platform^39^ integrated with the AlphaFold2 algorithm.^40^ The prediction was performed with default settings using the primary FASTA sequence as the query. Postprediction, ChimeraX-1.3 software was employed for processing the 3D structure (Figure 1B).

**Figure 1:**
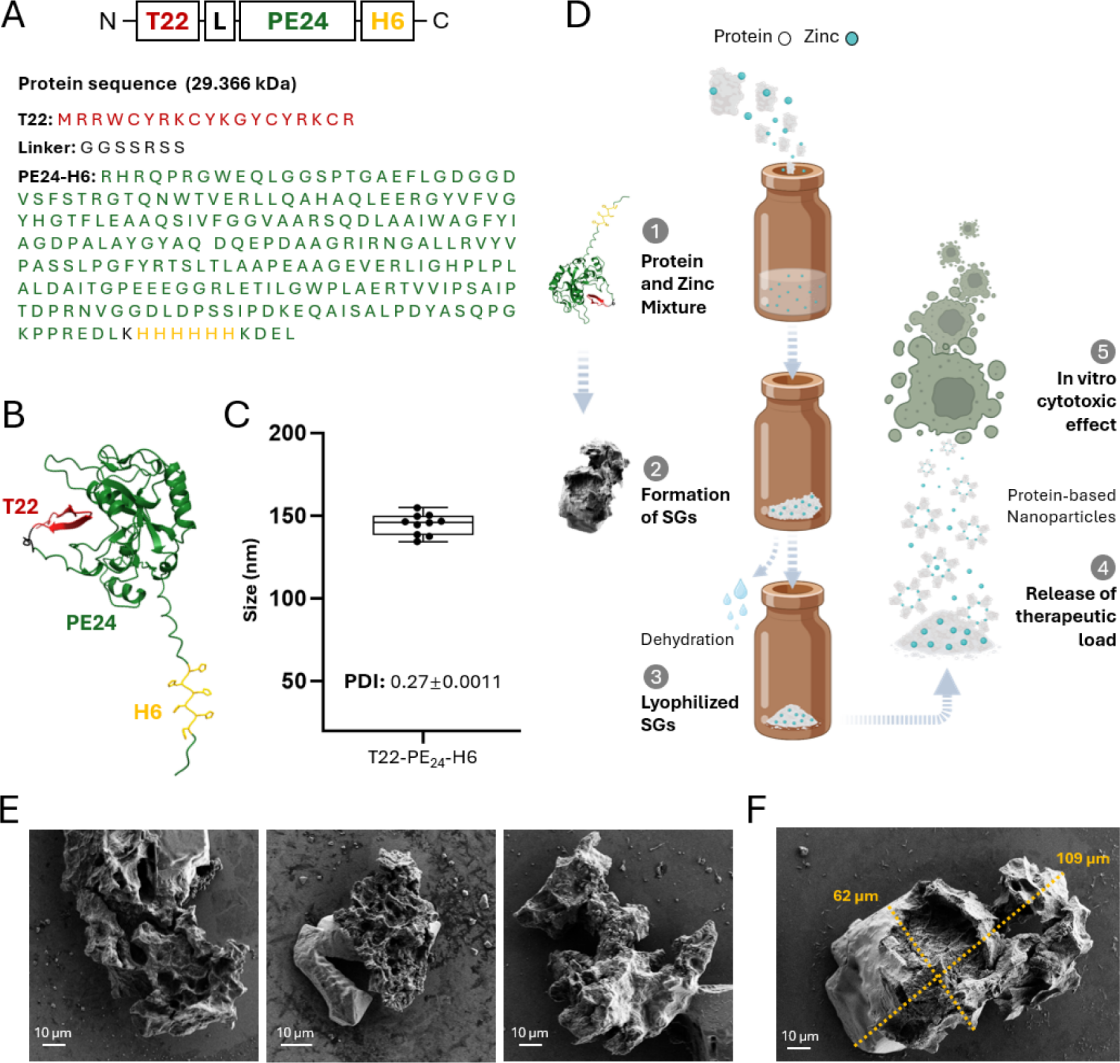
Preparation of T22-PE24-H6 and of secretory granules. (A) Amino acid sequence of T22-PE24-H6, indicated for each individual module. (B) A model of a monomer, obtained by AlphaFold. (C) Hydrodynamic size distribution and polydispersity index (PDI) of soluble T22-PE24-H6 upon chromatographic purification, measured by DLS. (D) Schematic and stepped representation of zinc-mediated formation of secretory microgranules. The formulation is intended for lyophilization, subsequent characterization and testing for targeted cytotoxic effects on CXCR4^+^ cells. The structural representation highlights key elements of the protein construct. (E) Microscopy images of randomly selected freshly obtained T22-PE24-H6 secretory granules taken by FESEM. Scale bars correspond to 10 μm. (F) FESEM image of a representative secretory granule, where scale bar was used to determine the granule size through ImageJ software.

### 2.3 Preparation of Secretory Microgranules and Lyophilization

The secretory microgranules were manufactured following protocols previously described,^31^ using ZnCl_2_ as a source of cross-linking, divalent cations (Zn^2+^) to interact with histidine residues in the recombinant protein His-tag. More specifically, the reaction used a molar 1:300 ratio of protein:cation and allowed for the precipitation of secretory granules, which were isolated by centrifugation (10 min, 10,000 × *g*), removing the soluble fraction. After preparation, all secretory granules were stored at −80 °C, without the addition of any buffer or storage solution. Control granules (specified as C in figures) were maintained at −80 °C and directly thawed ahead of use. Both WB and Bci conditions refer to lyophilized granules; therefore, they were thawed in preparation for lyophilization. WB granules were lyophilized without adding any solutions and without performing any additional steps. The Bci granules received the addition of 100 μL of citrate buffer (20 mM citrate pH 6.0 + 6% trehalose + 0.04% polysorbate) before lyophilization. All samples were prepared for lyophilization in a biosafety cabinet, with 1.5 mL Eppendorf tubes covered with Parafilm pierced 6–8 times per tube. Then, granules WB and Bci were frozen for 2 h at −80 °C, before being placed in a lyophilizer LyoQuest (Telstar) previously stabilized for temperature and vacuum conditions. The process was carried out for 16 h under vacuum levels lower than 0.05 mbar. Granules C, as a control, were not lyophilized. These granules were intended for further analysis of protein release aiming at cytotoxic effects in cell culture.

### 2.4 Size Measurement by Dynamic Light Scattering

Size measurements of the intensity size distribution of T22-PE24-H6 were performed by dynamic light scattering (DLS) at 25 °C and 633 nm in a Zetasizer Pro Blue (Malvern Instruments Limited). All samples were measured in 5 replicates. PDI values are expressed as the mean and standard deviation among the replicates.

### 2.5 Amyloid Detection Using Thioflavin (ThT)

Samples, including granules and soluble protein, were prepared at a final protein concentration of 0.1 mg/mL with 25 μM ThT in PBS 1× (pH 7.4 or pH 6, respectively) by mixing 100 μL of protein solution (1 mg/mL), 890 μL of PBS 1×, and 10 μL of diluted ThT (0.8 mg/mL). Fluorescence emission spectra were recorded from 470 to 600 nm with excitation at 450 nm using a Varian Cary Eclipse spectrofluorometer. A maximum emission peak near 482 nm indicated the presence of amyloid structures.^41^ pH 6 was used for the overall analysis of amyloid detection across all samples (meaning Sol, C_ThT_, C SGs, and Bci_20 mM_). An additional control of secretory granules at pH 7.4 was included for comparison purposes.

### 2.6 Electron Microscopy

For high-resolution electron microscopy, drops of 10 μL of each sample at 0.3 mg/mL were deposited on silicon wafers (Ted Pella, Inc.) and air-dried overnight. The images of the secretory microgranules, lyophilized or not, were obtained by using field emission scanning electron microscopy (FESEM Zeiss Merlin) operating at 2 kV and equipped with a high-resolution secondary electron detector. Representative images were obtained at a wide range of high magnifications. In one representative image, the size of the secretory granule was estimated using ImageJ software and employing the scale bar as a reference.

### 2.7 Analysis of Degradation and Release of Soluble Protein from Secretory Microgranules

Quantification of protein release from secretory granules was performed in triplicate using a spectrophotometric method. Granules were resuspended in 250 μL of sodium hydrogen carbonate solution (nonlyophilized C and lyophilized WB granules) or Milli-Q water (lyophilized Bci granules). Since lyophilized Bci granules (containing both protein and buffer salts) were reconstituted with the same volume of water as the original sample before lyophilization, these hydrated samples remained in citrate buffer 1×, in which further release experiments were performed. All of these resuspended granules were then incubated at 37 °C without agitation for periods varying from 3 to 30 days. At each time point, samples were briefly centrifuged to collect all drops from the lid, and granules were resuspended by pipetting up-and-down 10 times. 50 μL were taken from each sample and centrifuged for 10 min at 15,000 × *g* at 4 °C to isolate soluble and insoluble fractions. Soluble protein was then quantified in triplicate in a NanoDrop One System (Thermo Scientific) by measuring the absorbance at 280 nm. A theoretical extinction coefficient of 45,630 M^–1^ cm^–1^, calculated using the ProtParam software and assuming all cysteine residues form cystines in aqueous solvent, was applied. For panels where the release is expressed in percentage, all values obtained were normalized to percentage in reference to the total protein initially incorporated into the secretory granule (which corresponds to 100%). For degradation assays, aliquots were collected from granules incubated at 37 °C and analyzed by SDS-PAGE without separating soluble and insoluble fractions, to determine the degree of protein integrity at each time point. The SDS-PAGE was performed in triplicate in separate acrylamide gels, and a representative image of each condition is presented here.

### 2.8 Cell Culture and Cell Viability Assays

CXCR4^+^ cervical cancer cell lines (HeLa ATCC–CCL-2) were used as targets to determine the toxic activity of secretory granules in vitro. Cells were routinely cultured in Minimum Essential Medium (Mem Alpha Medium 1× + GlutaMAX, Gibco), supplemented with 10% fetal bovine serum (FBS, from Gibco) and incubated under a humidified atmosphere at 37 °C and 5% CO_2_. Viability assays were performed in opaque-walled 96-well plates using 3,500 cells per well, which were maintained for 24 h until 70% confluence was reached. Then, granules and soluble proteins were added in different concentrations and incubated for 48 h, and the cell viability was measured following the manufacturer’s instructions of CellTiter-Glo Luminescent Cell Viability Assay (Promega), measured in a Multilabel Plate Reader Victor3 (PerkinElmer). IC_50_ values were calculated using GraphPad PRISM 8.0.2. In addition, the internalization specificity through the CXCR4 receptor was tested by exposing cells to the CXCR4 antagonist AMD3100 ^42^ 1 h prior to protein incubation at a 1:10 ratio (protein/AMD3100) and following the previously described methods to determine cell viability.

### 2.9 Statistical Analysis

An initial assessment of normality and log-normality was performed using Shapiro-Wilk tests to verify the data’s normal distribution. Parametric data were analyzed through either one-way or two-way ANOVA or *t*-tests, depending on the number of groups and conditions. Nonparametric data were analyzed using the Kruskal–Wallis test. All measurements were conducted in triplicate or more, with peak values reported as mean ± standard error (SE). Statistical significance was indicated (*) at *p* < 0.05, (**) at *p* < 0.01, (***) at *p* < 0.001, and (****) at *p* < 0.0001.

## 3 Results

T22-PE24-H6 ^38^ is a modular protein that contains the exotoxin A of Pseudomonas aeruginosa (PE24),^43^ flanked by an 18-aa CXCR4-targeting peptide T22 and the hexa-histidine (H6) (Figure 1A), located immediately before the C-terminal subcellular targeting tetrapeptide KDEL (required for PE24 intoxication). Both T22 and H6 peptides are solvent-exposed in the folded protein, thus ensuring molecular cross-reactivity (Figure 1B). In solution, H6 promotes the oligomerization of the fusion protein in the form of stable nanoparticles around 150 nm (Figure 1C), whose formation is favored by divalent cations from the media that coordinate with the imidazole ring of His residues.^19–21^ T22 is a polyphemusin II-derivative peptide that specifically binds the chemokine receptor CXCR4 and that was discovered during anti-HIV drug exploration.^35^ T22, as a PE24 fusion, targets the whole nanoparticles to CXCR4^+^ metastatic cancer stem cells overexpressing CXCR4 for their selective destruction in vivo,^44^ as a highly potent but selective antitumor drug. T22-PE24-H6 protein nanoparticles were used as a model for testing the buffer composition and monitoring of nanoparticle release from reconstituted granules according to a stepped procedure (Figure 1D).

Secretory T22-PE24-H6 granules were formed by Zn-mediated protein precipitation and were visualized by FESEM as amorphous, insoluble materials of around 60–100 μm in size (Figure 1E,F). This type of material is termed a control in any further experimental process (Figure 2A). Alternatively, granules were lyophilized afterward, either without further processing (here referred to as WB – without buffer, since they did not receive any buffer before lyophilization) or lyophilized in the presence of citrate buffer (here named Bci). More specifically, before lyophilization, Bci granules were briefly resuspended in 20 mM citrate, previously determined as a good protein stabilizer during lyophilization of amyloids,^31^ thus rendering ready-to-use materials. Upon further reconstitution for functional and structural testing (Figure 2A), we first determined the amount of full-length T22-PE24-H6 in the samples at extended incubation times, using diverse control materials as described in Section 2 (Figure 2A). The proteolytic stability in the presence of citrate was excellent, as no protein degradation was observed for up to 30 days (Figure 2B). In contrast, significant spontaneous hydrolytic degradation occurred in soluble protein (Sol), in nonlyophilized granules (C), and in granules lyophilized in the absence of citrate (WB) (Figure 2B). These observations indicated not only that the granular version of T22-PE24-H6 was more stable than the soluble version but also that lyophilization in the presence of citrate positively contributed to preserving the proteolytic stability of the material (comparison of WB with Bci, Figure 2B).

**Figure 2:**
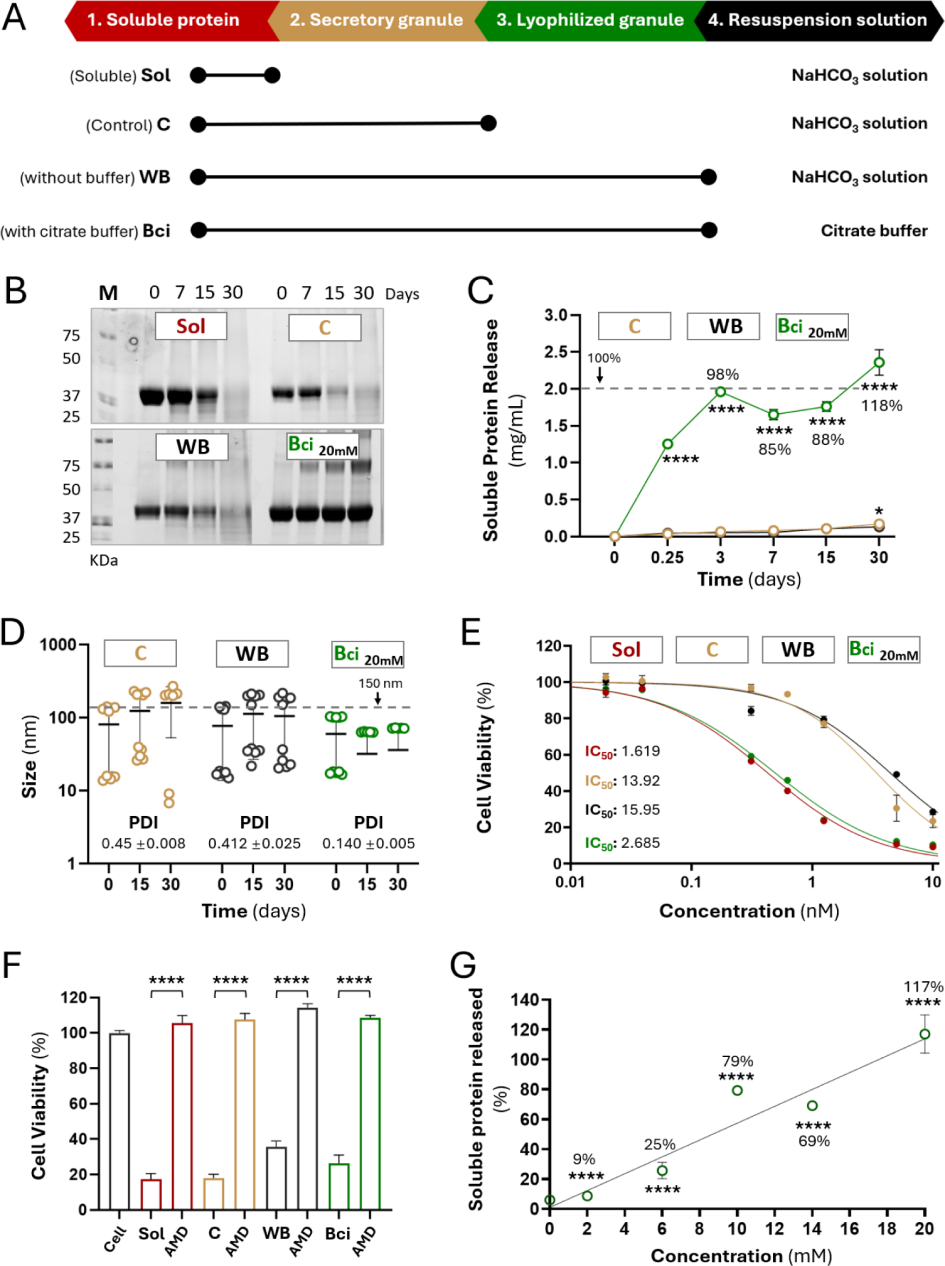
Protective effect of formulation on secretory granules. (A) Schematic representation of the experimental procedure focusing on control materials. (B) Evaluation of protein degradation in granules incubated for 30 days at 37 °C in different solutions, assessed by SDS-PAGE. (C) Soluble protein released from granules incubated at 37 °C in different solutions, measured along 30 days. The stippled line represents 100% release. (D) Hydrodynamic size of T22-PE24-H6 released from granules incubated at 37 °C in different solutions, measured along 30 days. PDI values were measured on day 30. The stippled line refers to 150 nm. (E) HeLa cell viability when exposed for 48 h to T22-PE24-H6 granules resuspended in different solutions and soluble protein. Viability was measured at different concentrations and IC_50_ value was determined for each condition. (F) HeLa cell viability and CXCR4-specificity of T22-PE24-H6. Cell survival was quantified after 48 h incubation in the presence of 10 nM of granules or soluble protein, using AMD3100 as an antagonist. (G) Soluble protein released from nonlyophilized granules incubated at 37 °C for 3 days. Nonlyophilized granules were resuspended in different citrate concentrations or in similar conditions of the control to determine the effect of citrate buffer on protein release, plotted as a linear regression (*R* ^2^ = 0.924). In the graph, 100% corresponds to the concentration of protein initially used to form the secretory granules (SGs). Data are expressed as mean ± SEM, *n* = 3 (at least). Statistical comparisons in relation to time 0 (panels B and C; ****p < 0.0001 or *p < 0.05), and/or AMD3100 presence (panel E; ****p < 0.0001). In panel G, all statistical comparisons were in relation to Bci 20 mM (****p < 0.0001).

The capability of all these materials to release protein (the functional purpose of the granular depots) was determined within 30 days under physiological conditions. Bci materials released significant amounts of protein already at day 3, reaching at that time a pseudoplateau (Figure 2C). Only limited amounts of protein were released from this point on. WB and C were also able to leak protein but in much lower amounts, which we presumed might be insufficient to enable them to act as drug delivery systems unless very low protein amounts are needed for a biological effect.

In all cases, the leaked material occurred as nanoparticles of around 100 nm (Figure 2D), slightly smaller than the starting building blocks (nanoparticles of around 150 nm; Figure 1C). This difference agreed with recent findings describing transitions of protein structure within functional secretory amyloids fabricated in vitro.^15^ The size reduction was particularly evident when citrate buffer was used in the formulation (Figure 2D), since the size of the leaked materials moved slightly below 100 nm in all of the tested samples and incubation times. Additionally, citrate buffers reduce the polydispersity index of the released nanoparticles, homogenizing the size in this particular condition. In the presence of sodium hydrogen carbonate solution (granules C and WB), the polydispersity index was much higher (Figure 2D). The final size of nanoparticles from citrate-based SGs (Bci), around 80 nm, has been found to be optimal for the uptake of nanoparticles upon exposure to cells.^45^ This is especially true if, as in the case of multimeric T22-PE24-H6 nanoparticles, they expose multiple cell ligands on the surface (here T22) in a virus-like fashion,^46^ thus allowing for cooperativity in the multiple binding to cell surface receptors^47^ and superselectivity in the cell targeting process.^48^ The enhanced compactness of the granules promoted by citrate might result from structural rearrangements occurring in them that would stabilize the protein and, at the same time, might assist in its release in the form of smaller nanoparticles. In this context, structural rearrangements in natural amyloids have also been observed, and a temporal progression toward more compact, dense, crystal-like structures has been documented for granular depots of both insulin^12^ and growth hormone.^7^

From the data shown here, we also noted that citrate contributed positively not only to modulating protein organization as nanoparticles but also to granule disintegration and consequent detachment of the oligomeric building blocks, making it possible to envisage a drug delivery platform. While citrate buffers present a lower pH than sodium hydrogen carbonate (pH 6 against pH 8), previous studies have already described that its influence on protein release is independent of pH and that other buffers with pH 6 fail to induce a similar effect.^31^ At this point, we were pushed to test the functional quality of the released materials through their cytotoxicity and cell selectivity through exclusive binding to CXCR4. For that, CXCR4^+^ HeLa cells were exposed to granular depots from which functional protein was expected to be released. As observed (Figure 2E), Bci granules caused cell death at levels comparable to soluble protein, with IC_50_ values within the same range and far from the nonreleasing equivalent materials (C and WB). All the T22-PE24-H6 materials selectively killed HeLa cells through CXCR4, as in all cases, the CXCR4-specific antagonist AMD3100 ^49^ inhibited nearly 100% of the killing process, irrespective of the extent of cytotoxicity/release (Figure 2F).

The above results were highly positive regarding microscale granules that could be used as slow drug delivery systems for therapeutic proteins or protein nanoparticles. However, the testing conditions, in which citrate has been shown as relevant, promoted a fast protein release that, after 3 days, essentially exhausted most of the protein available in the depot (Figure 2C). Since citrate is a positive promoter of protein release, we wondered if using concentrations below 20 mM could allow for progressive regulation of the delivery rate. In this regard, we confirmed that from 20 mM to at least 2 mM citrate, the amount of released protein decreased in a dose-dependent way (Figure 2G).

This observation opened a door to effectively exploring citrate as a regulator of protein release from artificial amyloids intended as slow protein delivery systems. For that, we tested the release kinetics, supramolecular organization, and functionality of the protein released from granules resuspended in three citrate concentrations below 20 mM (Figure 3A, left). The original amyloid architecture of SGs was conserved in citrate buffer, as confirmed by a ThT binding assay (Figure 3A, right). As expected, the protein release kinetics were positively influenced by citrate concentration (Figure 3B), and lowering the citrate content in the used buffer allowed for a more sustained release. Interestingly, the free solubilized nanoparticles were structurally more consistent at a high citrate concentration (Figure 3C). Indeed, the dispersion in the sizes of the T22-PE24-H6 nanoparticles released from C granules and those from granules intervened with 8 mM citrate must be noted. Using citrate at 11 mM or 14 mM, the nanoparticle size remained stable around 100 nm (Figure 3C) irrespective of the incubation time, indicative of robustness in the secretory materials and the secretory processes. Also, at these doses, the whole supramolecular constructs were more stable regarding proteolysis than those manipulated in the absence of citrate or using low concentrations of this agent (Figure 3D). In agreement with this statement, the materials released from granular depots involving 11 and 14 mM citrate were more monodisperse at each sampled time than the rest of the samples, as observed through their polydispersion indexes (Figure 3E). In agreement with the distinguishable release kinetics (Figure 3B) and differential structural stability of emitted nanoparticles (Figure 3C–E), the granular depots manipulated with 11 and 14 mM citrate, following a previous incubation at 37 °C in the absence of cells (the treatment shown in Figure 3D), were those rendering a clearer and more efficient cytotoxic effect on the in vitro HeLa cell model, indicating resistance to degradation or inactivation (Figure 3F). These results suggest that by packaging proteins as granules, the cytotoxic activity is retained for a longer time than without packaging, thereby enabling a sustained cell-killing capacity over extended periods.

**Figure 3:**
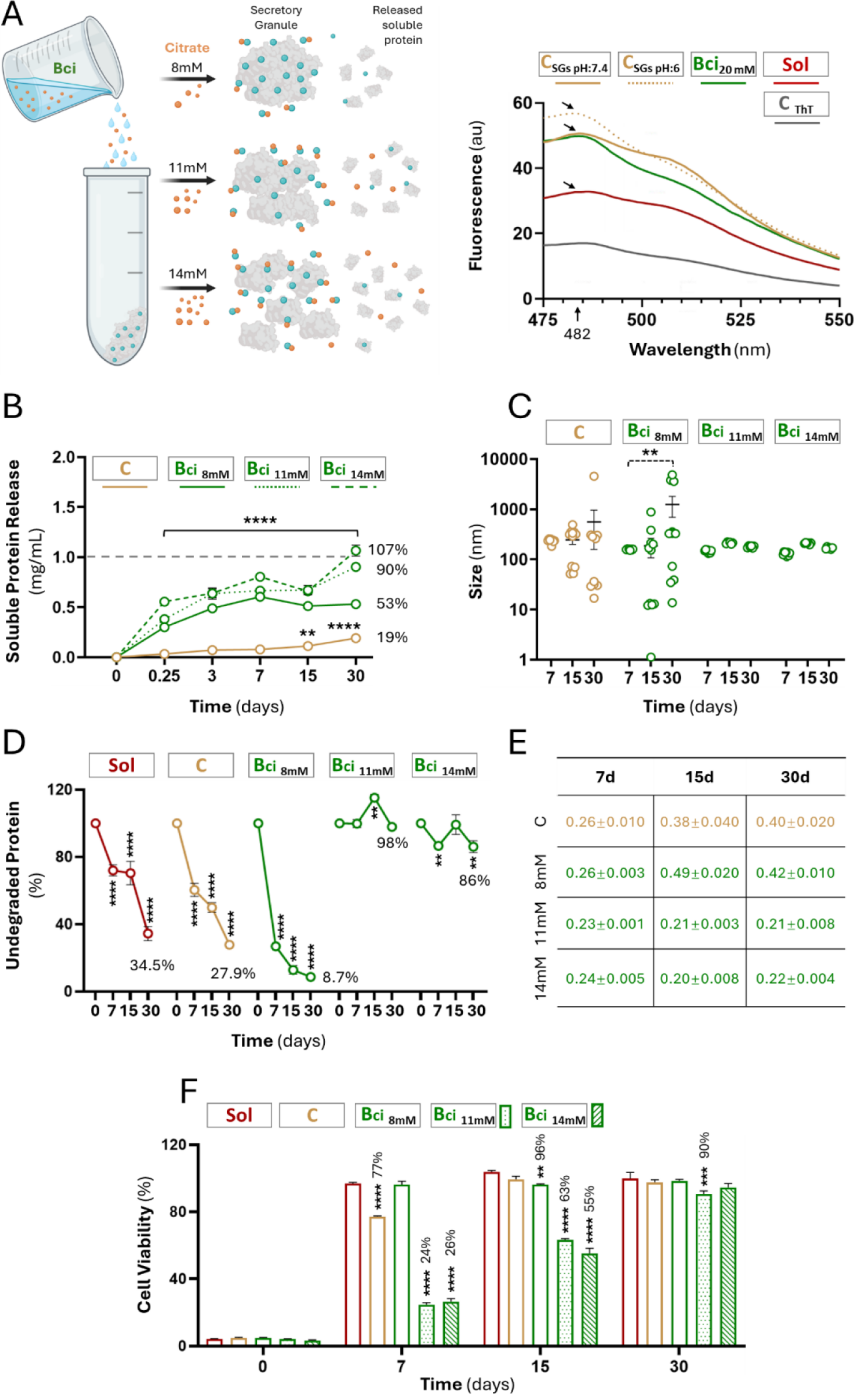
Role of citrate on the secretory function. (A) Left: schematic representation of the different concentrations of citrate assessed. Right: a fluorescence emission profile illustrating the incubation of SGs with Thioflavin T (ThT) to detect the presence of amyloid structures. ThT exhibits a maximum emission peak of approximately 482 nm in the presence of such structures (indicated by arrows). *C*_ThT_ refers to the ThT sample without protein. Sol refers to the soluble T22-PE24-H6 protein in the presence of ThT. B) Soluble protein released from granules resuspended in different solutions and incubated at 37 °C for up to 30 days. (C) Hydrodynamic size of T22-PE24-H6 released from granules incubated at 37 °C in different solutions, measured along 30 days. (D) Evaluation of protein remaining in granules resuspended in different solutions and incubated for 30 days at 37 °C, assessed by SDS-PAGE. (E) PDI values of T22-PE24-H6 released from granules incubated for 30 days at 37 °C. (F) Evaluation of the cytotoxic potential of T22-PE24-H6 granules incubated over a 30-day period at 37 °C in different solutions and tested for cytotoxicity at different time points. HeLa cell viability was measured after 48 h exposure to T22-PE24-H6 granules. Data are expressed as mean ± SEM, *n* = 3 (at least). Statistical comparisons in relation to time 0 (panels B and D; ****p < 0.0001 or **p < 0.01); in relation to time 7 (panel C; **p < 0.01), and in relation to soluble sample (in red) in each time point (panel F; ****p < 0.0001, ***p < 0.001, **p < 0.01).

## 4 Discussion

As with other proteins previously tested for Zn-assisted SG fabrication, the presence of H6 in T22-PE24-H6 enables this protein to self-assemble into large aggregates,^18^ whose formation requires H6 but no other protein segments (such as T22).^18^ In this Zn-His aggregation platform, SGs occur as a dead end of a process that renders progressively complex protein networks, in which oligomerization depends on the presence of H6.^17,18^ Then, the formation of relatively stable early oligomers is inhibited by soluble His but not by other amino acids.^19^ In this context, the set of data generated in the present study validates the artificial SGs (Figure 1D,E) as a slow delivery system suited for protein-only nanoparticles, intended for a biological action. Zn-mediated protein precipitation, lyophilization, storage, and further reconstitution (Figure 1A) do not impair the capability of the microscale granules to leak the building block, which in the model T22-PE24-H6 (Figure 1B), is an oligomeric structure of around 150 nm (Figure 1C). The manipulation of the protein during granule fabrication and use also preserves the capacity for oligomerization of T22-PE24-H6 (Figure 1C), its capacity to selectively bind cells through CXCR4 (Figure 2F), and the cytotoxic potential of the bacterial toxin (Figure 2E). When formulated with 20 mM citrate, most of the protein content is released in about 3 days (Figure 2C), whereas formulating with either 8, 11, or 14 mM citrate extends this protein exhausting period to 30 days (for 14 mM), a little bit longer (11 mM), or a significantly extended period (for 8 mM, Figure 3B). On the other hand, short-term citrate leaking from citrate-based SGs was detected by ^1^H NMR spectroscopy (not shown). It must be noted that in this self-contained protein depot platform, in which heterologous holding materials are avoided, protein release occurs through the disassembling of the SGs, and that all their structural components or potentially retained excipients are released to the media together with the protein. In this context, the Zn content in SGs in an injectable formulation has been calculated to be far below the FDA-recommended dietary doses.^20^ Also, the concentration of citrate upon reconstitution of the lyophilized product is also estimated to be below that of many FDA/EMA-approved drugs such as rituximab and biosimilars.^50,51^ It must be noted that citrate (E331) is a common excipient in commercial protein drug formulations^52^ (apart from a regular food additive). This fact guarantees the safety of the platform if citrate is seen as convenient for incorporation to SG preparation when aiming at specific applications in which the protein drug release should be adjusted.

Several clinical contexts demand time-prolonged drug-releasing systems,^53,54^ many of them involving protein drugs.^55^ The artificial SG platform proposed here might contribute as a preferable alternative to other approaches, in which the protein drug is embedded in a holding matrix, device, or container formed by nondrug materials.^56^ Once subcutaneously implanted, these SGs release stable oligomeric nanoparticles whose multimeric organization benefits from virus-like properties, such as enhanced interactivity with target cells or preserved biological activity.^46^ The administration or implantation of polymers, metals, lipids, ceramics, and other materials as drug holders not only increases the complexity of fabrication but also poses toxicity concerns.^57^ Artificial SGs are self-contained, self-delivered protein depots that fulfill the rising demand of “taking the vehicle out of drug delivery”,^58^ since the drug itself is progressively detached from the particles (formed exclusively by the drug) as they disintegrate by the physiological chelation of Zn. Looking at the complexity of finding the right dose and temporal profile in the treatment of, for instance, rare diseases,^59^ or as recently stated, in vaccination,^60^ citrate, as a formulation component, is shown here as a critical modulator of the protein detachment kinetics. The correct determination of proper citrate amounts (when necessary) might allow for tailoring of SGs for specific vaccination or therapeutic settings, with different requests regarding the kinetics of drug or antigen availability.

Citrate has been observed not only as a modulator of protein leakage rate but also as a potent protein stabilizer that preserves the integrity of polypeptides over time (Figures 2B and 3D). Also, this molecule, as a component of granule formulation, mediates conformational modifications in the forming polypeptides that rearrange them into different-sized nanoparticles, moving from 150 nm (Figure 1C) as the starting material to 100 nm as the acting drug (Figure 3C). Such citrate-dependent structural modification is also reflected by the enhanced repeatability and reproducibility of size measurements when analyzing the output material (Figures 2D and 3C), minimizing nanoparticle size dispersion. While structural transitions between *in* (forming) and *out* (released) material have already been identified,^15^ citrate is clearly assisting in stabilizing them. Among the pleiotropic activities shown by citrate,^61^ such a chaperone-like performance is in agreement with its known interactions with proteins that stabilize them.^62,63^ Structural stabilization of both proteins and protein oligomers combined with a faster leakage of nanoparticles from citrate-based SGs can synergistically account for the more pronounced cytotoxicity of T22-PE-H6 compared to alternative SG versions formulated in the absence of citrate (Figures 2E and 3F).

In this regard, adding citrate to protein formulations offers advantages in mitigating protein degradation and providing a controlled release of protein from secretory granules (Figure 3B,D). This efficacy as a chelating and stabilizing agent is probably coupled with its role in maintaining colloidal stability^64^ by modulating the balance of electrostatic, *H*-bonds or hydrophobic interactions among protein molecules. Structurally, citrate is an α-hydroxy-polyanionic carboxylate molecule^65^ with two negative charges and one hydroxyl group that introduces one potential *H*-bonding site per molecule (Figure 4A). These features enable citrate to interact effectively with positively charged residues on protein surfaces (modeled here with T22-PE24-H6, Figure 4B), stabilizing these interactions and reducing electrostatic repulsion. This reduction in repulsion, as well as stabilization on the protein surface,^64,66^ suggests a mitigation in protein–protein interactions that could otherwise lead to aggregation,^66^ thereby preserving a stable colloidal state.^67^

**Figure 4:**
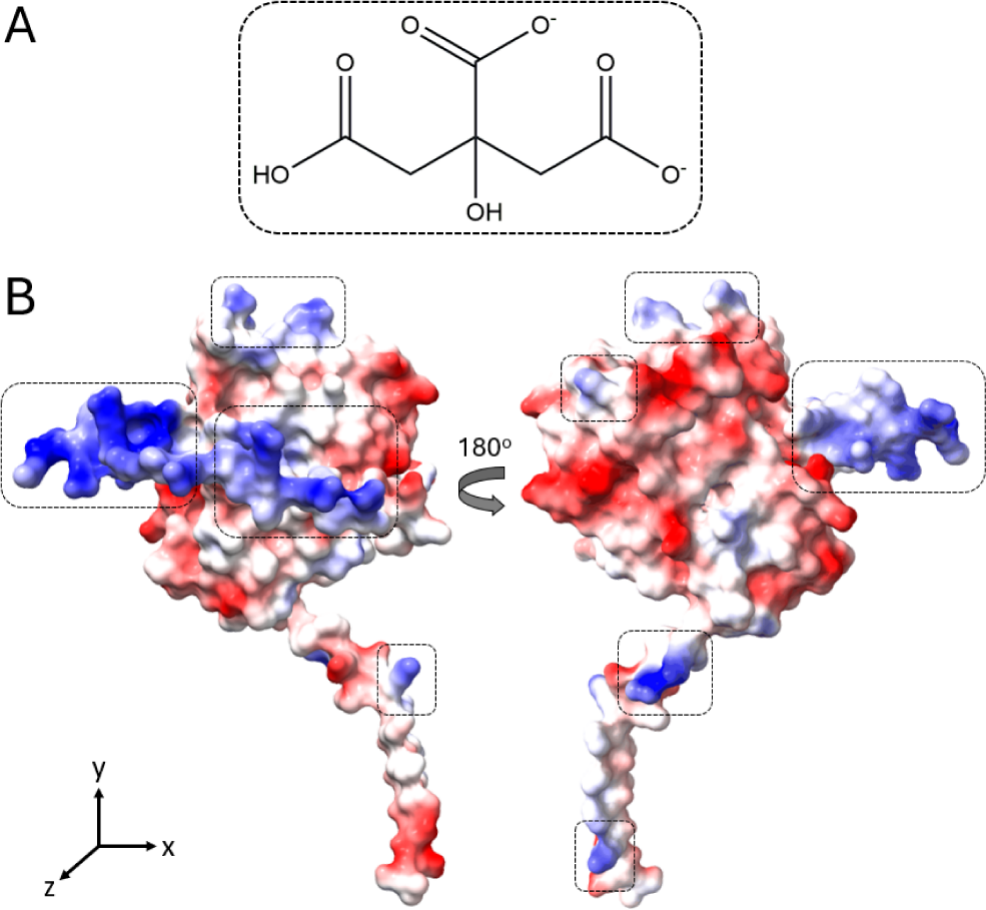
Hypothetical supramolecular interactions between citrate and T22-PE24-H6. (A) Schematic representation of molecular structure at pH 6. (B) Surface charge distribution of T22-PE24-H6 displayed in the *xz* plane (left) and *xy* plane (right), with negatively charged residues shown in red and positively charged residues in blue. Potential interaction regions between the protein and citrate are highlighted with dotted squares.

Furthermore, citrate provides stability against environmental stressors, such as pH fluctuations,^68^ by buffering the solution and maintaining proteins in their native conformations. This stabilization may reduce susceptibility to common degradation pathways,^69^ including both aggregation and some degradation mechanisms. Moreover, citrate, as a chelating agent,^65,69^ can sequester metal ions that can catalyze oxidative reactions, another factor contributing to protein degradation and colloidal destabilization. Additionally, citrate’s influence on protein solubility and colloidal behavior can impact the release kinetics of protein-based drugs. By forming complexes with proteins,^66^ citrate supports a slow and sustained release profile, where its interactions can modulate the diffusion rate of proteins from its formulation as secretory granules. It is also observed that in lyophilized formulations,^70^ citrate plays a role in stabilizing protein phases, facilitating slow reconstruction and sustaining protein activity over extended periods of time.

In our protein platform, whose capability to be finely regulated in vivo must still be explored, the presence of citrate allows for the complete (or almost complete) disintegration of the material under physiological conditions (Figures 2C and 3B), progressively. This capability to promote disassembling involves the totality of aggregated proteins, depending on the citrate dosage. This is in contrast with the behavior of citrate-free granules, which only release a fraction of embedded protein, as an important part of their content is reluctant to disintegrate.^29^

## 5 Conclusion

In summary, the inclusion of citrate in the formulation of synthetic secretory granules enhances, as expected, protein stability and improves the structural reproducibility of the oligomers (observed as a strong reduction of size polydispersion) that are released during the endocrine-like leaking process. Furthermore, citrate allows for variable protein release velocities in a concentration-dependent manner (Figures 2G and 3B). In comparison with citrate-free materials, in which protein release is modest and materials formulated with 20 mM citrate that disintegrated in around 3 days, the use of intermediate citrate concentrations allows for a dose-dependent regulation of protein release. Such a simple approach, whose fine-tuning would need to be further confirmed in clinical settings, offers a versatility of this synthetic dynamic protein depot platform so far unexpected, that allows for its application as a slow-delivery system to be adapted to fulfill specific biomedical demands.

## Data Availability

https://doi.org/10.34810/data1850
